# Interfacial Adsorption of Oil-Soluble Kraft Lignin and Stabilization of Water-in-Oil Emulsions

**DOI:** 10.1021/acs.langmuir.3c03950

**Published:** 2024-02-29

**Authors:** Jost Ruwoldt, Berhane Handiso, Marianne Øksnes Dalheim, Amalie Solberg, Sébastien Simon, Kristin Syverud

**Affiliations:** † RISE PFI AS, Høgskoleringen 6B, NO-7094 Trondheim, Norway; ‡ Ugelstad Laboratory, Department of Chemical Engineering, Norwegian University of Science and Technology (NTNU), NO-7491 Trondheim, Norway

## Abstract

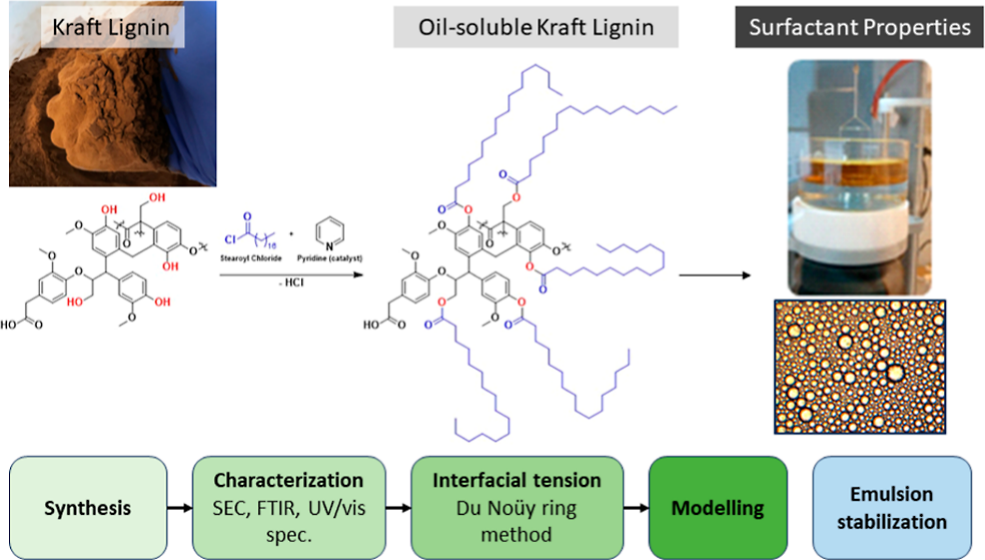

In this paper, the potential of esterified Kraft lignin as a novel oil-soluble surfactant was examined. The lignin was chemically modified by esterification with lauric or stearic acid, making it soluble in solvents such as toluene or *n*-decane. Adsorption at the oil–water interface was then studied by the Du Noüy ring-method. The oil-soluble lignin behaved similar to water-soluble lignin surfactants, both the qualitative and quantitative progression of interfacial tension. Modeling revealed a surface excess of 7.5–9.0 × 10^–7^ mol/m^2^, area per molecule of 185–222 Å^2^, and a diffusion coefficient within the range 10^–10^ to 10^–14^ m^2^/s; all of which are in line with existing literature on water-soluble lignosulfonates. The data further suggested that the pendant alkyl chains were extended well into the paraffinic solvent. At last, bottle tests showed that the oil-soluble lignin was able to stabilize oil-in-water emulsions. The emulsion stability was affected by the concentration of lignin or NaCl as well as the oil phase composition. Aromatic oils exhibited lower emulsion stability in comparison to the aliphatic oil. In conclusion, a new type of surfactant was synthesized and studied, which may contribute to developing green surfactants and novel approaches to valorize technical lignin.

## 1 Introduction

Lignin is a polyaromatic branched biopolymer that is commonly found in wood and other lignocellulose biomass. Due to its chemistry and structure, lignin exhibits distinct features, which delineate lignin from common polysaccharidic biopolymers such as cellulose, starch, and chitin. Extensive research has been conducted to develop new application areas for lignin, such as polymeric materials, platform chemicals, carbon materials, and specialty chemicals.^1−4^ While there are a multitude of promising technologies under development, the largest use of lignin for value-added purposes remains that of water-soluble lignin dispersants. The objective of this article was hence to synthesize and study a new type of lignin surfactant, oil-soluble Kraft lignin.

Lignin is generated during biosynthesis from the parent compounds *p*-coumaryl- (*p*-hydroxyphenyl, H-unit), coniferyl- (guaiacyl, G-unit), and sinapyl alcohol (syringyl, S-unit).^5^ These monolignols differ in the existence of zero, one, or two methoxy groups ortho to the hydroxyl group. The monolignols are further cross-linked via various carbon–carbon and carbon–oxygen linkages, hence yielding a randomly branched “near-infinite” network. During pulping or biorefinery operations, both the aryl-alkyl and the β-O-4 bonds are most prone to cleavage.^6^ At the same time, new carbon–carbon linkages can be formed.^7^ Technical lignin, i.e., the lignin-rich feedstock produced by pulping and biorefinery operations, hence tends to carry a greater abundance of condensed structures than natural lignin. In addition, other functionalities can be added depending on the isolation process, which include carboxylic and sulfonic acid groups. In their salt form, the latter two can contribute to the water-solubility of lignosulfonates, depending on the counterion of choice.^8,9^ Technical lignin may be distinguished based-on the isolation process, yielding various types such as Kraft lignin, lignosulfonates, soda lignin, and hydrolysis lignin.^10^ The final structure and composition is defined by the isolation, purification, and potential chemical modification, which in turn defines the physicochemical properties of the lignin surfactants.^11^

Adsorption of water-soluble lignin onto solid surfaces reportedly follows the Langmuir isotherm.^12−15^ The adsorption process in affected by a variety of parameters, including concentration, salinity, pH, and the water- and oil-phase composition.^9,16^ In solution, the lignin macromolecules were found to exhibit ellipsoidal shape and self-associate on the flat edges into planar agglomerates.^17−19^ Aggregation of lignosulfonates in aqueous solution can occur at concentrations as low as 0.05 g/L.^20^ Hydrophobic interactions and π–π stacking were pointed out as the main mechanisms governing such aggregation.^18,21^ Water-soluble lignin can furthermore form viscoelastic interface layers, which can exhibit gelling properties in the presence of multivalent cations.^22^ Stabilization of oil-in-water emulsions is achieved by a number of mechanisms, including steric hindrance, the Marangoni–Gibbs effect, electrostatic repulsion, viscoelastic interface layers, and particle stabilization.^23^ While a combination of effects usually occurs, the dominant stabilization mechanism depends on the composition and aggregate state of the lignin. For instance, water-soluble lignin can act by interfacial adsorption and electrostatic repulsion at sufficiently large charge density,^24^ whereas water-insoluble lignin may form particle stabilized Pickering emulsions.^25^

Chemical modification of lignin has been researched for a variety of purposes. In surfactant chemistry, the modulation of physicochemical properties is often the goal,^11^ whereas other approaches may seek to enable or improve the use in polymeric materials.^26,27^ Concerning the latter, increasing the reactivity of lignin is frequently the goal, which can be achieved by alkylation (epoxy resins),^28^ allylation or alkynylation,^29,30^ phenolation (phenolic resins),^31^ or carboxylation (polyesters).^27^ Water-solubility for the use as surfactants is usually facilitated by adding ionizable moieties, e.g., by sulfonation,^32^ sulfomethylation,^33^ or carboxylation.^34^ The opposite effect, i.e., hydrophobization and lipophilization, can be achieved by silylation or esterification with fatty acids.^35,36^ Such treatments have also been demonstrated to improve compatibility with olefin thermoplastics, e.g., polyethylene or polypropylene.^26,37,38^ It has also been demonstrated that Kraft lignin esterified with fatty acids was soluble in nonpolar solvents.^39^ Esterification of technical lignin with fatty acids can be done via reactive intermediates, i.e., chlorinated fatty acids or anhydrides.^36,37,39^ Alternatively, the phenolic hydroxyl groups were converted into aliphatic ones first, which provided better reactivity, hence enabling a direct approach by reacting the hydroxyl groups with carboxylic acids at elevated temperatures.^40,41^ Still, while there is a great abundance of modifications proposed for technical lignin, the usefulness of such methods in an industrial setting is often overlooked.

Solubility of lignin in organic solvents is important to process, modify, and utilize this biopolymer.^27,42^ Technical lignin, such as Kraft, soda, and organosolv lignin, is reportedly soluble in some aprotic polar solvents, such as dimethyl sulfoxide and dimethylformamide, and some protic polar solvents, such as ethylene glycol, ethylene glycol monoethyl ether (“Cellosolve”), 1-methoxy-2-propanol (“Dowanol”), and diethylene glycol monobutyl ether (“Butyl Carbitol”).^43,44^ Both the extraction process and the biomass origin can have minor effects on the solubility of these lignin types. Lignosulfonates, on the other hand, are highly hydrophilic and are hence reported to be soluble in water, propylene glycol, ethylene glycol, and dimethyl sulfoxide.^45^ The solubility of lignin may change as a result of chemical modification or fractionation. Lower molecular weight fractions of the lignin are usually more soluble in solvents, in which the whole lignin sample is not considered soluble. Duval et al. utilized this principle to fractionate Kraft lignin into molecular weight fractions with lower polydispersity.^46^ Thielemans and Wool further reported that esterification with short-chain alkyl chains rendered the Kraft lignin more soluble in less polar solvents.^47^ Longer chains accounted for a greater shift in the Hansen Solubility Parameter, where the butyrated sample was soluble in styrene. Still, lignin esterified with alkyl-chains longer than four carbon atoms is currently addressed very little in the literature. So, despite the fact that grafting with long alkyl-chains can render lignin compatible with nonpolar solvents, there is currently a lack of comprehensive characterization of the physicochemical properties of such chemistries.

Eco-friendliness is one advantage of lignin esterified with fatty acids, as the ester bond is hydrolyzable, and the fatty acids are considered biodegradable. Several authors have thus addressed both synthesis and application of such chemicals.^26,36,40^ By the introduction of alkyl moieties, the lignin is rendered highly hydrophobic. There are thus reports, which have employed esterified lignin as barrier coatings for cellulosic materials.^48^ Esterification of Kraft lignin has furthermore been shown to improve compatibility with olefinic thermoplastic polymers, such as polyethylene or polypropylene.^26,37,38^ Composite films and coatings of esterified lignin and poly(lactic acid) have also been reported.^49^ In addition, esterified Kraft lignin was dissolved in light gas oil and hydrotreated to produce green diesel fuel.^39^ Interestingly, there is even one report, which utilized lignin esterified with long chain fatty acids as a stabilizing agent for oil-in-water emulsions.^50^ However, the stabilization mechanism was of Pickering type, indicating limited or no solubility in the oil phase (toluene). Procedures for producing oil-soluble lignin have hence been developed, yet very little attention has been paid to the surfactant use of these. The suitability of water-soluble lignin for surfactant applications would suggest that oil-soluble lignin may be just as useful, e.g., in applications such as drilling mud dispersants and agrochemical formulations, which are oil-based. Still, there is a lack of studies investigating the surfactant properties of such a lignin. This work therefore summarizes our efforts to study the interfacial activity and emulsion stabilization properties of Kraft lignin, which had been rendered oil-soluble by esterification with lauric or stearic acid. A synthesis based on esterification with fatty acids was chosen as Kraft lignin possesses an abundance of hydroxyl groups for this reaction. In addition, the ester bond is hydrolyzable and fatty acids are considered biodegradable, hence producing a product that would be more accessible for biodegradation than other types of modifications.

## 2 Experimental Section

### 2.1 Materials

Kraft lignin was supplied as BioPiva 395 by UPM Biochemicals (Germany/Finland), toluene (99.8%, anhydrous), dimethylformamide (DMF, 99.8%, anhydrous), dimethyl sulfoxide (DMSO, ≥99.9%, ACS reagent), *n*-decane (≥95%), 2-methyl tetrahydrofuran (MeTHF, ≥99.5%, ReagentPlus), Cellite 535 filtration aid (SiO_2_, particle size 0.02–0.1 mm), and lauroyl chloride (98%) were purchased from Sigma-Aldrich (Norway). Pyridine (≥99.7%, analytical reagent for KarlFischer titration) and stearoyl chloride (≥97.0%, TCI Europe) were obtained from VWR (Norway). Ethanol (99.7%, technical grade) and 2-propanol (99.5%, technical grade) were acquired from KiiltoClean (Norway). Petroleum distillate was purchased as Blåtind White Spirit from Wilhelmsen Chemicals AS, (Norway). Fumed silica (commercial name AEROSIL 200) was supplied by Evonik Industries (Germany). Distilled water was further purified via a Milli-Q system from Millipore to a resistivity of 18.2 MΩ × cm.

### 2.2 Sample Preparation

The Kraft lignin was alkylated by esterification using a modified version of the procedure by Hult at al.^36^ Five grams of Kraft lignin was first dried under vacuum at 55 °C for 5 h. The lignin was then dissolved in 30 mL of DMF and 2.55 mL of pyridine. To this solution, 8.0 mL of lauroyl chloride was added to obtain the C_12_ esterified Kraft lignin. Alternatively, 7.28 g of stearoyl chloride was dissolved in 40 mL of toluene and added in two steps to obtain C_18_ esterified Kraft lignin. The reaction was conducted under a constant stream of nitrogen for 20 h and ambient conditions. The product was purified by quenching in water, which also removed DMF and pyridine, followed by extraction with ethanol to remove toluene. The residual viscous liquid was then blended with Cellite filtration aid and extracted with ethanol or water (pH 7 buffered with phosphoric acid/sodium hydroxide) in the case of C_12_ esterified Kraft lignin, or 2-propanol in the case of C_18_ esterified Kraft lignin. The modified lignin was finally recovered via extraction with toluene, filtration of the solution through a glass-fiber filter, and solvent removal by vacuum distillation and finally evaporation at 55 °C under a constant stream of nitrogen. The final yield was approximately 7–9 g of modified lignin. This yield shows that a mass increase was noted, as should be after grafting with fatty acids, and that most of the esterified lignin was recovered.

Derivatization of the Kraft lignin was necessary to provide a reference during size-exclusion chromatography (SEC) since the lignin raw material was not soluble in the MeTHF solvent. Butyration of Kraft lignin was hence performed by weighing 0.4 g of dry lignin into a vial and adding 4 mL of pyridine and 6.92 mL of butyric anhydride. The vial was sealed and stored under ambient conditions in a moisture-free environment for 20 h. The butyrated lignin was recovered by quenching in ice-cold water, filtration through a 0.1 μm filter, washing the filter cake with 200 mL of water three times, drying in air, and evaporation in vacuum at 55 °C for 5 h. The procedure yielded approximately 0.5 g of butyrated lignin.

Prior to interfacial tension measurements, the petroleum distillate was purified of residual surfactants. 300 mL of petroleum distillate and 0.5 g of fumed silica (Aerosil 200, Evonik Industries, Germany) were added to the glass bottle. The dispersion was shaken overnight at 145 rpm with an IKA HS 501 Digital horizontal shaker (IKA-Werke, Staufen, Germany). Then, 35 mL from the mixture was carefully transferred to a 45 mL centrifugal tube. Centrifugation was performed with an Eppendorf Centrifuge 5810 (Eppendorf, Hamburg, Germany) for 5 min at 7000 rpm and only the top 20 mL (supernatant) was collected for subsequent interfacial analysis.

### 2.3 Characterization

SEC was conducted on an Agilent 1260 Infinity II system with refractive index (RI) detection. One PLgel 5 μm Mixed-D 300 × 7,5 mm column with a separation range of ca. 200–400,000 Da was operated at 22 °C and a guard column in front. MeTHF was used as the mobile phase at a flow rate of 1 mL/min. Calibration was done using eight polystyrene (PS) standards from the Shodex SM-105 kit, whose logarithmic molecular weight to retention time profiles were fitted with a third-degree polynomial. Lignin samples were prepared at 5 g/L in MeTHF solvent with injections of 50–100 μL per sample run. As a reference, the blank sample was butyrated as described in Section 2.2, as the pristine or acetylated lignin were only partly soluble in MeTHF. The number (*M*_n_)- and weight (*M*_w_) average molecular weights were calculated from the PS-calibrated data according to eqs 1 and 2, where *N*_*i*_ denotes the molar concentration (RI-detector signal divided by *M*_*i*_) and *M*_*i*_ the PS-calibrated molecular weight at data point *i*.
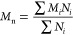
1
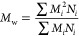
2

UV/vis spectrophotometry was conducted on a Shimadzu UV-1900 UV–vis spectrophotometer according to our previously published procedure.^43^ Each spectrum was recorded from 500 to 250 nm (DMSO) or 200 nm (decane) at 1.0 nm intervals and medium speed. Stock solutions of 0.2–0.5 mg/mL lignin in blank solvent were made and measured within 24 h of preparation. For each run, 200–600 μL of stock solution were pipetted into the quartz cuvette and diluted with 1600–2700 μL blank solvent. The volumes were adjusted to yield an absorbance of 0.3–1.0 cm^–1^ at 280 nm. Samples were run in duplicate with two dilutions, yielding four measurement points per sample. The absorptivity was computed by dividing by the sample concentration (dry mass).

Fourier-transform infrared (FTIR) spectroscopy was conducted on a PerkinElmer Spectrum 3 with a transmission module. Kalium bromide (KBr) was dried in an oven at 120 °C and kept in dry storage when not in use. KBr pellets were pressed with 0.5 wt % lignin for each analysis. The background spectrum was recorded on blank KBr pellets. The analysis went from 500–4000 cm^–1^ in 4 cm^–1^ increments, measuring 64 scans and two runs per sample. Each spectrum was baseline-corrected by a manually fitted cubic spline. Normalization was additionally done by dividing through the aromatic stretching band at 1508 cm^–1^.

### 2.4 Interfacial Tension Measurement

Interfacial tension measurements were performed using a Sigma Attention 701 instrument (Biolin Scientific AB, Gothenburg, Sweden) equipped with digital control and data acquisition. A platinum Du Noüy Ring was used. Each sample was prepared immediately before the interfacial measurement, as previous results have indicated that aging may affect the sample.^43^ The modified lignin was weighed into a volumetric flask and topped up with petroleum distillate, and the mixtures were stirred until complete dissolution. At last, the solutions were sonicated using an Elma S 30 H Elmasoic (Elma Schmidbauer, Singen, Germany) sonic bath for 15 s in the degassing mode. The ring and the glass vessel were rinsed thoroughly with water, acetone, toluene, and acetone, followed by water at last, and dried. The platinum ring was furthermore cleaned in a gas flame for 5 s. For each interfacial tension analysis, 40 mL water (heavy phase) were added into the vessel, followed by 40 mL of lignin solution (light phase) on top. The ring was placed right below the water–oil interface before the measurement. Experiments were run for 5 h. The average of two to three independent measurements was reported for each data point. The interfacial tension between pure water and the purified petroleum distillate was used as the reference.

### 2.5 Emulsion Stabilization

Modified lignin was dissolved in the oil phase as described above. Fifteen milliliters of purified water and 10 mL of oil phase were pipetted into 45 mL centrifugation tubes and emulsified at 20,000 rpm for 1 min using an ULTRA TURRAX T 25 fitted with 18 mm head from IKA Werke GmbH & Co. KG (Germany). The emulsions were stored overnight under ambient conditions and thereafter centrifuged at 5000 rpm for 5 min (ThermoFisher Scientific Heraeus Multifuge X3). The aqueous phase at the bottom of each vial was subsequently removed via needle and syringe, collected, and weighed, yielding the amount of free water. By dividing through the amount of water that was initially added, the free water percentage was calculated. The type of emulsion (water-in-oil) was confirmed by adding a drop of densely packed emulsion to each of the two phases.

## 3 Results and Discussion

### 3.1 Lignin Characterization

The modified lignin was characterized to confirm the success of the esterification reaction and to provide information about the product. Both C_12_ and C_18_ esterified Kraft lignin became soluble in less polar solvents, such as toluene, xylene, MeTHF, and decane. Here, solubility was visually confirmed by the brown color that the solvent attained. Since the pristine Kraft lignin was not soluble in these solvents, it can serve as a demonstration for successful alkylation.

**Figure 1:**
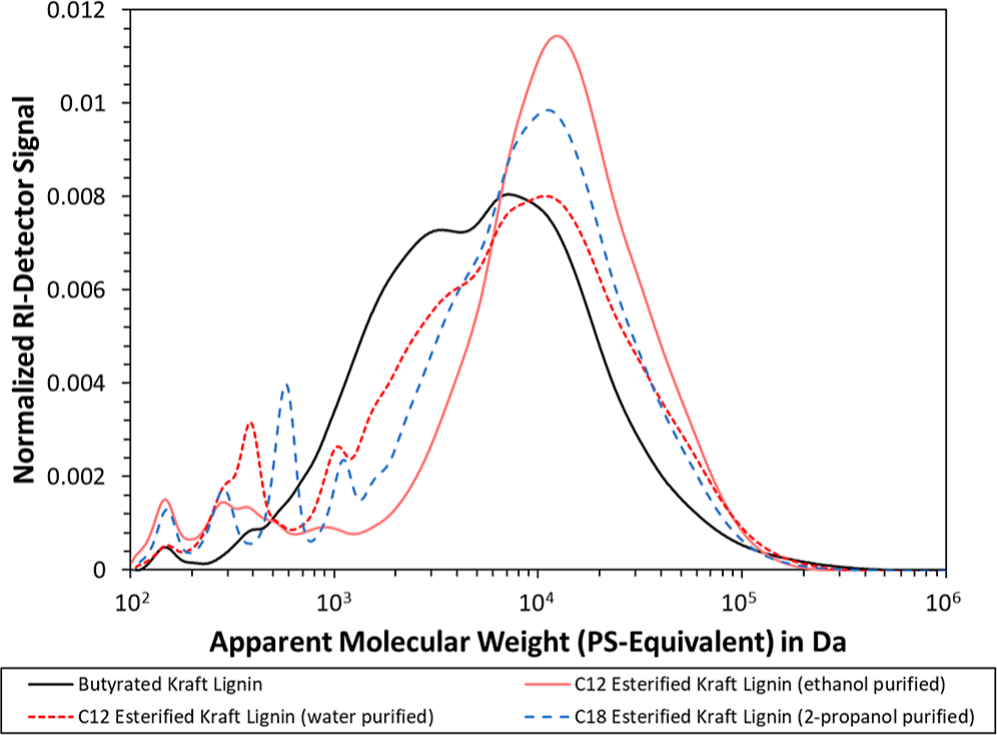
Apparent molecular weight distribution in PS-equivalent obtained by SEC of alkylated lignin samples in 2-methyl THF. All graphs were normalized by each peak area.

As grafting adds matter to the lignin macromolecule, the reaction should, in principle, yield an increase of molecular weight, which can manifest itself as an increase in the hydrodynamic volume. This change was investigated by SEC, as illustrated in Figure 1. The global maximum of the butyrated Kraft lignin indeed occurred at a lower molecular weight than for both C_12_ and C_18_ esterified Kraft lignin, hence indicating successful grafting. Interestingly, the purification method appeared to also affect the final product. As can be seen, C_12_ esterified Kraft lignin contained a greater amount of low-molecular weight components when purified with pH 7 buffered water as compared to ethanol. This would hence suggest that unreacted lauric acid was better removed by the alcohol than by water. In addition, it appears that low-molecular-weight fractions of the lignin were removed with ethanol, as the main peak started appearing only at 2000 Da, as compared to 700 Da for the water-purified sample. Purification of the C_18_ esterified Kraft lignin was consequently done with an alcohol, as this would yield a product with fewer monomer residues. The solubility of stearic acid is greater in 2-propanol, which was hence adapted instead of ethanol. As can be seen, the peak at 500–600 Da is still visible for C_18_ esterified Kraft lignin, which coincided with the elution of a pure stearic acid run. This suggested a low but significant amount of residual precursor in the lignin. It is in theory possible to improve purification further by choosing an alcohol with a higher amount of carbon chains; however, this would also remove more of the modified lignin, so 2-propanol was viewed as a good middle ground. Purification of the esterified lignin by the removal of residual reactant residues is important, as this improves the quality of both the characterization and interfacial measurement data in subsequent sections. The removal of low-molecular-weight compounds also decreased the polydispersity of the lignin, which is marked by a narrower main-peak. A decrease in polydispersity also makes sense, as the lignin macromolecules with a lower molecular weight tend to exhibit more reactive sites.^51^ More alkyl groups will therefore be added per lignin macromolecules, as compared to the lignin with a higher molecular weight. Interestingly, the butyrated Kraft lignin exhibited a longer tail at the high-end of the molecular weight. This trend is counterintuitive, as the C_12_ and C_18_ esterified Kraft lignin should always be greater. Still, the latter two are also extracted from the Cellite filtration aid by toluene, which necessitates the solubilization of all components in this solvent. It is plausible that some lignin remained on the Cellite since the particles were still not entirely, white after extraction despite the washing solvent being entirely clear. This residual lignin could then be favoring high-molecular-weight compounds, hence explaining the loss in high-end macromolecules. Another possible explanation is the degradation of lignin during the reaction. Despite using ambient temperature, the pyridine may act as a base to catalyze depolymerization reactions, which could, in turn, reduce the observed maximum molecular weight. The conformation of the lignin should be noted. Due to its polybranched nature, the lignin backbone cannot undergo steric reconfiguration to the same extent as linear polymers. The pendant alkyl chains, on the other hand, may curl up, hence diminishing the contribution by the pendant alkyl chain length.

Using the data in Figure 1, we calculated the apparent average molecular weights in Table 1 were calculated. Kraft lignin is reported to exhibit *M*_n_ and *M*_w_ values of approximately 1000–3000 and 1500–25,000 g/mol, respectively.^10,52^ These values closely align with the outcome in Table 1, hence corroborating the results. However, it has been argued that the comparison of linear polymers with the randomly branched lignin macromolecule are not entirely straightforward.^53^ For the same hydrodynamic volume, the molecular weight of branched polymers tends to be higher than for linear polymers, as the conformation is more compact.^54^ This would, in theory, yield an underestimation of the actual molecular weight of the lignin. The values in Table 1 may hence be interpreted with caution. Still, their potential for intersample comparison is unimpeded by this. As can be seen, the C_12_ esterified Kraft lignin exhibited a lower apparent *M*_w_-value after water purification as compared to ethanol, which is likely due to the removal of low molecular weight (macro-)molecules. The apparent *M*_w_ of C_18_ esterified Kraft lignin is lower than that of the C_12_ lignin after alcohol purification, which could again be due to a higher number of residual monomers. The polydispersity index (PI) is lower for the butyrated sample, whereas the esterified samples all range between 3.4 and 4.6. This highlights the contribution of monomeric residues to the polydispersity of the esterified lignin.

**Table 1:** Calculated Apparent Number Average (*M*_n_) and Weight Average (*M*_w_) Molecular Weight (g/mol), as Well as the PI

sample	*M*_n_ in PS equ	*M*_w_ in PS equ	PI
butyrated Kraft lignin	4000	10 900	2.7
C_12_ esterified Kraft lignin (water purified)	3500	14 000	4.0
C_12_ esterified Kraft lignin (ethanol purified)	3600	16 500	4.6
C_18_ esterified Kraft lignin (2-propanol purified)	4000	13 600	3.4

**Figure 2:**
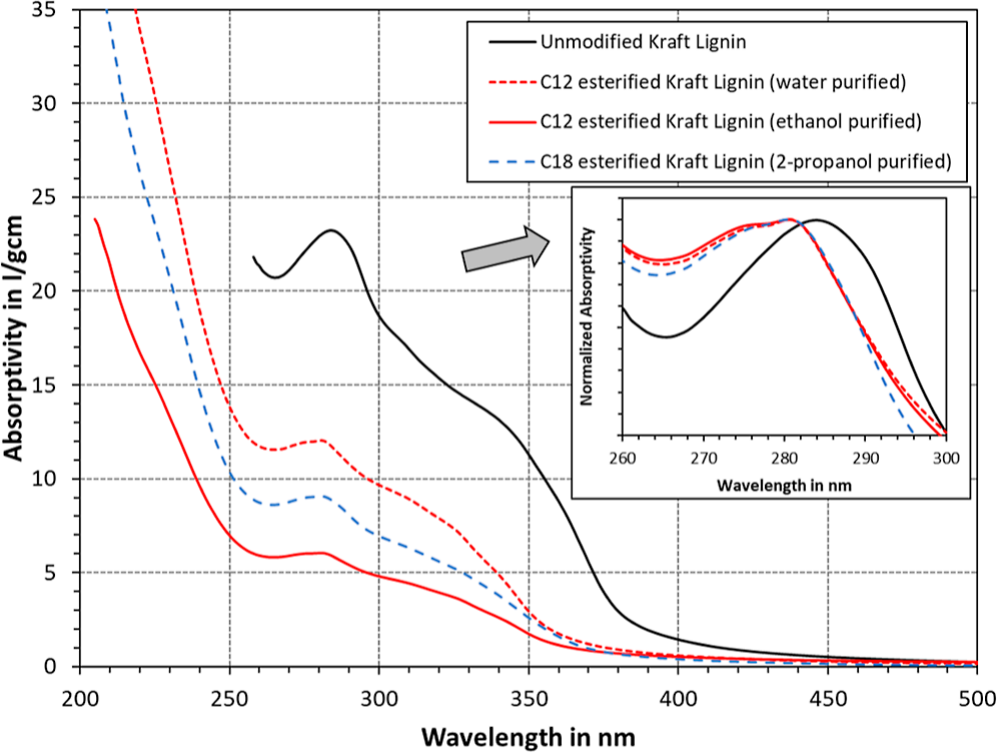
UV/vis photospectrometry of unmodified Kraft lignin in DMSO and alkylated Kraft lignin in a decane solvent.

Reactions with the phenolic hydroxyl groups in lignin can induce hypsochromic and hypochromic shifts in the UV-spectra.^55^ This was indeed also observed for the samples in this study, as illustrated in Figure 2. Compared to the unmodified lignin in DMSO, the esterified Kraft lignin in the decane solvent exhibited lower absorptivities (hypochromic shift). However, the addition of alkyl chains will also inevitably reduce the relative abundance of aromatic moieties per sample weight unit. It is therefore difficult to directly delineate this effect. The purification method appears to have a great effect on the absorptivity as well, since C_12_ esterified Kraft lignin showed lower values after ethanol purification than after using water. It would thus appear that the low-molecular-weight components have a great impact on the observed spectrum, which were removed during ethanol purification. Hypsochromic shifts, on the other hand, are not affected to the same degree. For better comparison, Figure 2 also shows the spectra normalized at a maximum around 280 nm. In each case, the spectra were shifted to the left after esterification (hypsochromic shift), which is evidence of successful chemical modification.^55^ In addition, the shift appeared to be the same for all modified samples, suggesting that the degree of esterification is approximately the same. At last, due to alcohol extraction yielding the purer product, the denotation “C_12_ esterified Kraft lignin” will be used in the subsequent text to refer to the C_12_ lignin purified by ethanol extraction.

**Figure 3:**
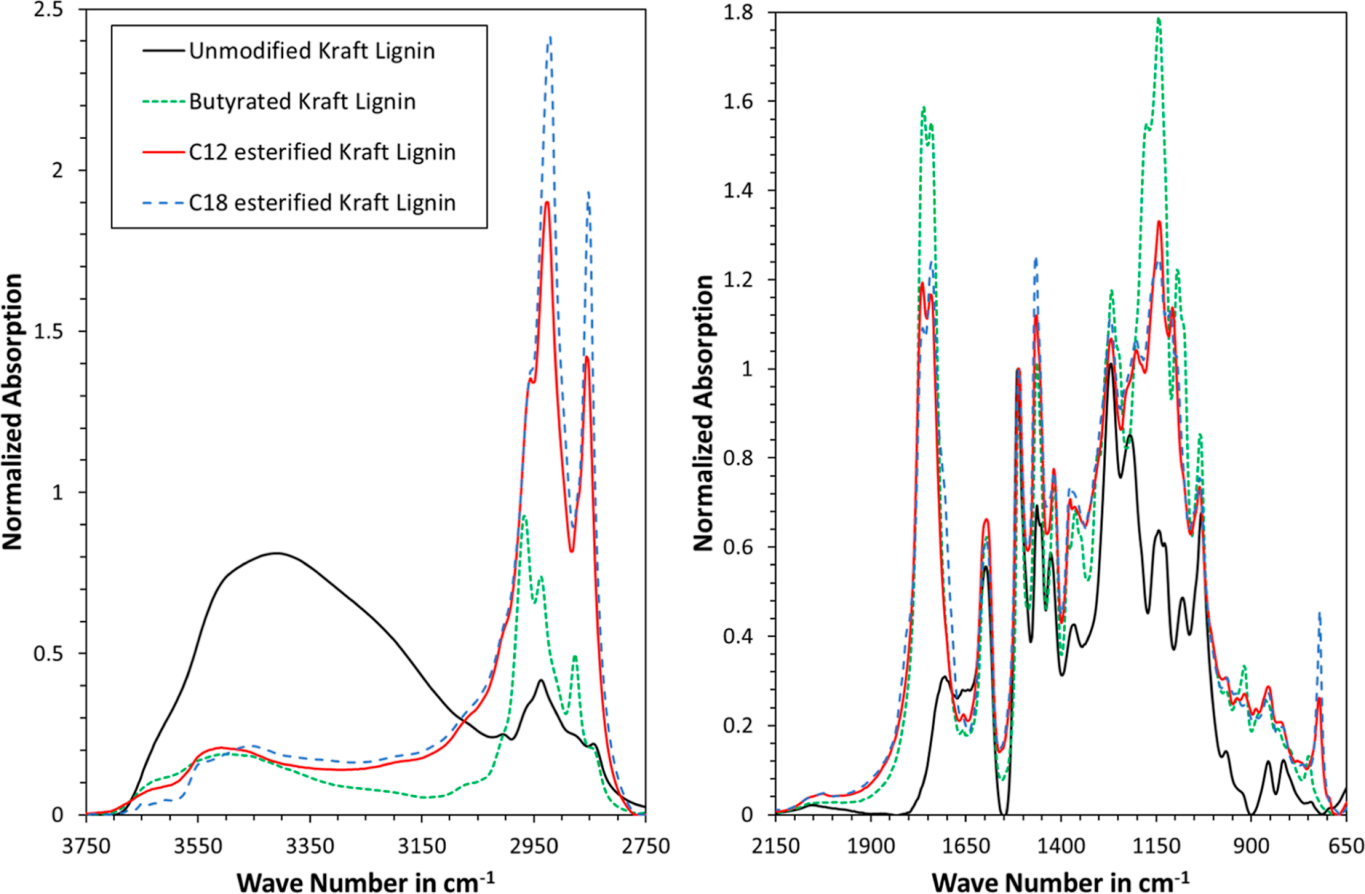
Transmission FTIR spectra of modified and unmodified lignin. (C) Mp = 0.14 equiv. The spectra were baseline-corrected with a cubic spline and normalized via an aromatic stretching band at 1505–1510 cm^–1^.

As the third technique, FTIR spectroscopy was used to characterize the products. Spectra were baseline-corrected and normalized at the aromatic stretching peak at 1508 cm^–1^, as this facilitates a better comparison. As can be seen in Figure 3, the unmodified lignin exhibited a broad peak at the OH-stretching around 3400 cm^–1^. This peak decreased in intensity and width after chemical modification, hence illustrating the conversion of OH-groups into ester bonds. A certain signal is still present after modification, which could be attributed to unreacted OH-groups, carboxylic acid groups in the lignin, and residual fatty acids. Conversely, the intensity of the carbonyl stretching band at 1750–1740 cm^–1^ was increased after modification, which confirms the success of the reaction. It is interesting to note that the carbonyl stretching of butyrated Kraft lignin was higher than that of the C_12_ and C_18_ esterified samples. Butyration followed the established procedure for acetylation, which assumes complete reaction of the OH groups in lignin. Based on this, it can be concluded that the degree of substitution for esterification was not complete but that both the C_12_ and C_18_ esterified Kraft lignin exhibited the same degree of esterification. The peak at 3000–2800 cm^–1^ corresponds to C–H stretching in the methyl and methylene groups. This peak greatly increased in intensity after modification, further underpinning the addition of alkyl chains. The intensity of C_18_ esterified Kraft lignin is greater than that for C_12_, the latter of which is in turn greater than the C–H stretching band of the butyrated lignin. Such order makes sense considering the length of the grafted alkyl chains. The fingerprinting region around 1800–800 cm^–1^ also changed in intensity and qualitative progression. Such changes would be evident, considering the introduction of alkyl moieties and the changes in the phenolic structures in the lignin. All in all, the FTIR results are in line with established literature,^37,56^ underpinning successful esterification of the Kraft lignin with fatty acids.

### 3.2 Effect on Interfacial Tension

**Figure 4:**
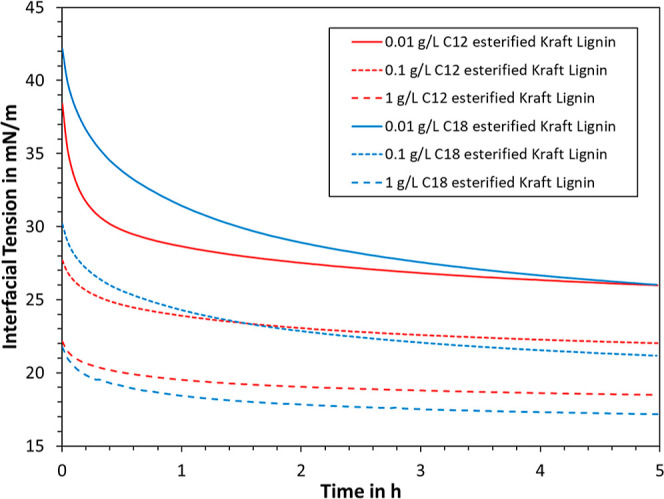
Time-dependent interfacial tension at different concentrations of modified lignin at the water/oil interface.

**Figure 5:**
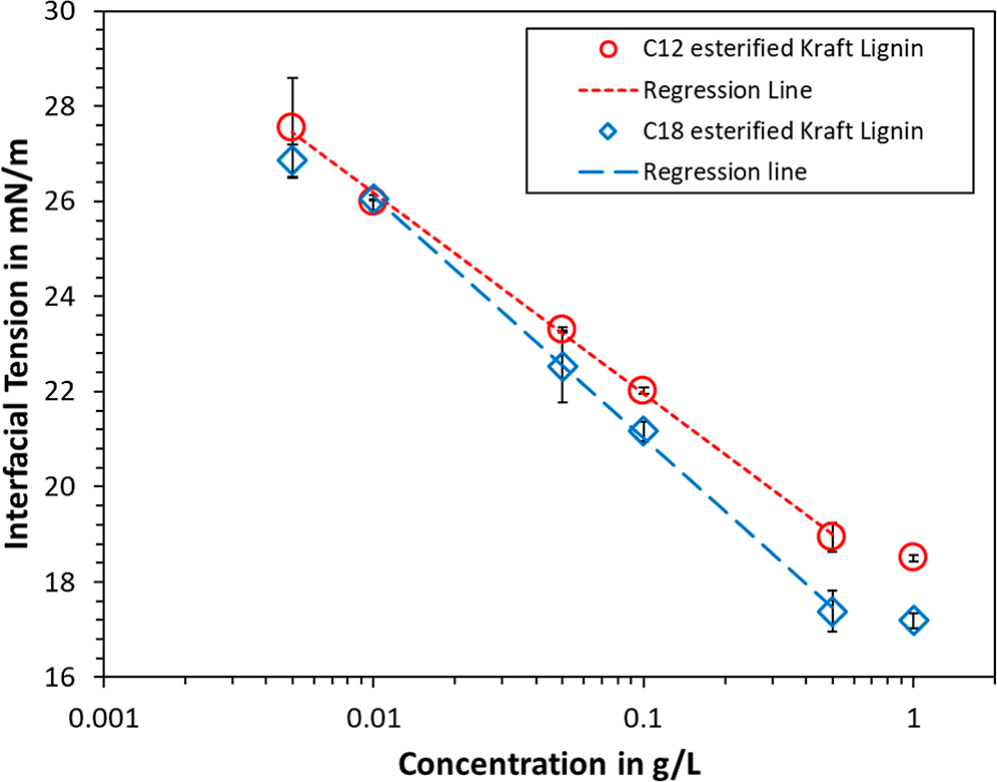
Dependence of the equilibrium interfacial tension on the concentration of modified lignin.

Adsorption of lignin at the water–oil interface is a kinetic process that can be traced by measuring interfacial tension over time. The experimental data are plotted in Figure 4, illustrating a sharp decrease in interfacial tension at the start of the measurement, followed by a plateauing and the approaching of a constant value toward later time units. The same qualitative progression has also been documented for water-soluble lignin surfactants, such as lignosulfonates and sulflonated Kraft lignin.^9,57^ Initially, the process is controlled by diffusion, with lignin macromolecules entering the interface. At longer times, diffusional exchange and steric restructuring are attributed for the slower changes in interfacial tension.^22,57^ The changes in interfacial tension are slower at lower lignin concentrations, likely due to a lower concentration in the bulk and therefore lower statistical probability that a lignin macromolecule is in the right position to undergo adsorption. At the final experiment time of 5 h, minor changes were still visible in the interfacial tension (Figure 4); however, existing literature suggests that even after several days of equilibration, the interfacial tension does still not converge at a constant value.^22^ Equilibrating for several days was considered experimentally unfeasible, as this could also promote effects such as solvent evaporation and chemical degradation of the modified lignin.^58^ A period of 5 h was hence taken by convention, which the same equilibration time used in other published works.^9,16,22,59^ The concentration levels in Figures 4 and 5 were chosen based on previous experience with water-soluble lignin surfactants, targeting the linear-logarithmic decrease in equilibrated interfacial tension with concentration.^9,22^ At the bottom line, it was found that the kinetic interfacial behavior of oil-soluble lignin surfactants is similar to that of their water-soluble counterparts.

The equilibrated interfacial tension (at 5 h) was plotted as a function of concentration in Figure 5. A linear logarithmic region is noticeable at concentrations 0.01–0.3 and 0.003–0.3 g/L for C_18_ and C_12_ esterified Kraft lignin, respectively. At low concentrations, the interfacial tension is reported to approach values of the surfactant-free case,^20,24^ i.e., 47.7 mN/m for the pure petroleum distillate. At concentrations above the linear-logarithmic regime, aggregation has been reported for water-soluble lignin surfactants.^24,60^ A similar observation was made for the oil-soluble lignin surfactants synthesized in this work, i.e., a deviation from the linear-logarithmic progression to a less pronounced decrease at higher lignin concentrations. This would hence suggest the advent of bulk aggregation within the region of 0.3–1 g/L.

### 3.3 Interfacial Modeling

The data in Figure 5 can, furthermore, be used to compute the Gibbs adsorption isotherm and other molecular properties. First of all, the slope of the linear-logarithmic region corresponds to the term  in eq 3,^61^ where γ corresponds to the interfacial tension and *c*_L_ to the lignin concentration. Using the temperature *T* = 22 °C, the ideal gas constant *R*, and *M*_n_ from Table 1 to calculate lignin molar concentration, the surface excess Γ was computed.
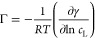
3

From the surface excess Γ and the Avogadrós constant *N*_av_, the area per molecule *A*_m_ was then computed via eq 4.^61^
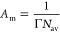
4

Using eqs 3 and 4, the surface excess and area per molecule were computed and are listed in Table 2. The values are in line with other results published in literature. For example, a surface excess of 4.6–7.5 × 10^–7^ mol/m^2^ and an area per molecule of 135–358 Å^2^ have been reported for lignosulfonates adsorbing at the water–oil interface.^9,22^ The area per molecule of C_18_ esterified Kraft lignin is slightly lower than that for the C_12_ lignin, which is likely related to a lower *M*_w_ value (see Table 1). The measured area per molecule is furthermore on the upper end of reported values for Gemini surfactants or asphaltene model compounds, which possess significantly lower molecular weight.^62,63^ This would thus suggest that mainly the low-molecular-weight fractions of the lignin are adsorbing and that the lignin is likely present as collapsed globules at the interface. Such an interpretation concurs with the fact that the lignin macromolecule is highly branched, which would promote the behavior of collapsed globules rather than extended linear polymers. Still, it should be noted that the data in Table 2 must be interpreted with caution. As evidenced by the SEC elution profiles, there are limited residues of fatty acids that could interfere with the interfacial tension measurement. Fatty acids adsorbing at the interface could provide lower interfacial tension values than those for the lignin itself, potentially yielding a lower area per molecule. Interactions with the esterified lignin are also possible, which could decrease the effect on interfacial tension, e.g., by sticking to the lignin and forming lignin-fatty acid aggregates. In addition, even at 5 h equilibration time, the interfacial tension was still decreasing for some of the measurements (see Figure 4). So strictly speaking, this would not allow the application of the Gibbs adsorption isotherm. At the same time, it is experimentally difficult, if not unfeasible, to measure the complete equilibration of lignin-based surfactants.^22^ The surface excess should hence be taken as an overestimate, while still being comparable to other experimental studies, as the same equilibration time was chosen by convention.^9,16,22,59^

**Table 2:** Average and Standard Deviations of Surface Excess and Area per Molecule

sample	surface excess, mol/m2	area per molecule, Å^2^
C_12_ esterified Kraft lignin	7.5 × 10^–7^ ± 0.5 × 10^–7^	222 ± 13
C_18_ esterified Kraft lignin	9.0 × 10^–7^ ± 0.4 × 10^–7^	185 ± 8

To determine the diffusion coefficient, the long-time approximation by Ward and Tordai was chosen.^64^ This approach proposes a model to describe the surface excess under the assumption of a diffusion-controlled process. It is furthermore assumed that enough time has passed for the subsurface to reach bulk concentration. The dynamic interfacial tension γ_dyn_ is then given as a function of the equilibrium interfacial tension γ_∞_, the concentration *c*_L_, the time *t*, and the diffusion coefficient *D*.
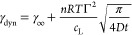
5

**Figure 6:**
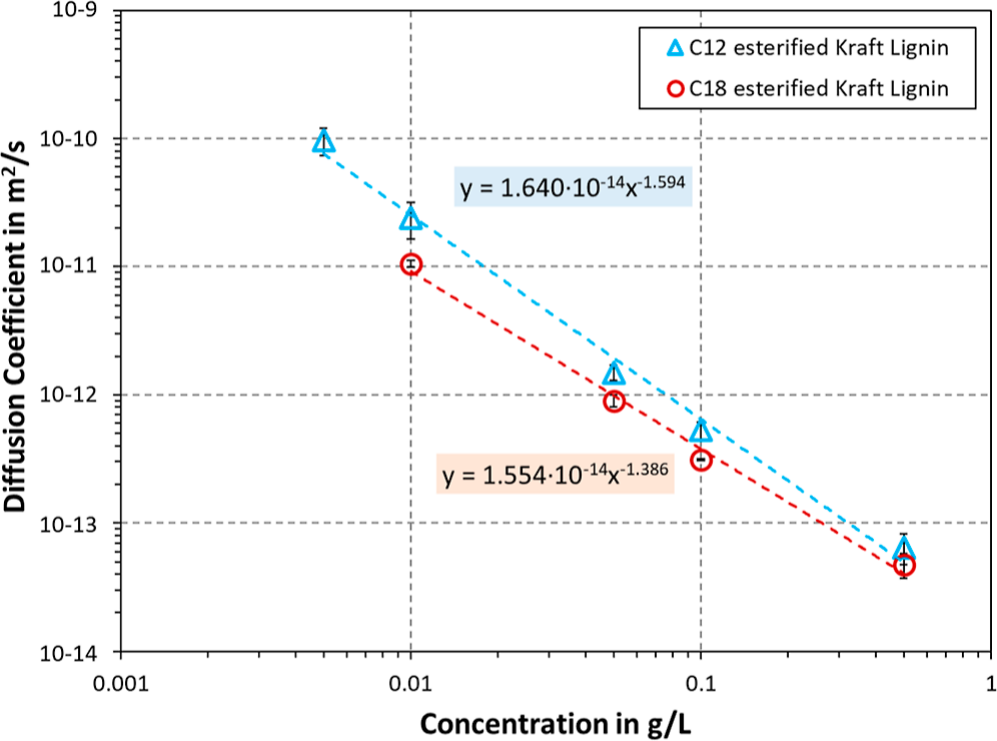
Diffusion coefficient as a function of the concentration of modified lignin in petroleum distillate. The dotted lines denote the log–log fit obtained from regression with eq 6.

Computationally, the data of the dynamic interfacial tension γ_dyn_ in dependence of  was fitted by a linear regression line at *t* → 5 h. The regression intervals were adjusted manually, computing the slope that equaled , and finally solving for *D*. The results are plotted in Figure 6. As can be seen, the diffusion coefficient showed decreasing values for increasing concentrations. The values for C_12_ esterified Kraft lignin were higher than those for C_18_, albeit the latter exhibiting a lower *M*_w_ value and both having similar grafting degrees. The macromolecules of C_18_ esterified Kraft hence appeared to move slower in the solvent than those of C_12_. This would indeed suggest that the alkyl chains are extended outward into the solvent, providing a higher hydrodynamic radius for macromolecules with longer pendant chains. In addition, it appears that the solvent greatly affected the conformation of the pendant alkyl chains. SEC utilized MeTHF, while also probing the hydrodynamic radius. It would only make sense that the change from MeTHF to a petroleum distillate affected the conformation of the pendant alkyl chains during the interfacial measurements, as the latter solvent included mainly C_10_ to C_13_ alkanes. Overall, the decrease in diffusion coefficient with concentration was linear for both macromolecules in the log–log chart. A similar behavior was observed for petroleum asphaltenes when studying interfacial dynamics.^65^ Sztukowski and Yarranton fitted this dependence by eq 6, which describes the diffusion coefficient *D* as a function of the concentration *c* and the fitting constants *a* and *b*.
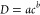
6

The fit from eq 6 was in good agreement with the experimental data. Three possible explanations can be put forward to explain this trend. First, this could suggest that esterified Kraft lignin is undergoing adsorption in the aggregated state. Higher concentration increases agglomerate size, hence, increasing the hydrodynamic radius and reducing the diffusion coefficient. This interpretation is in contrast to the interfacial tension results in Figure 5, which suggests aggregation only above 0.3 g/L. Still, bulk and interface aggregation remain two distinct phenomena. Second, the obtained trend could also suggest that the adsorption kinetics is not purely controlled by diffusion of lignin, and there exists an energy barrier to the adsorption. Third, there could be reorganization in the adsorbed layer, which would influence the interfacial tension kinetics.^66^ In both of the latter cases, this would result in diffusion coefficient data, which do not reflect the “true” values, meaning that no conclusion on the aggregation state of lignin in bulk could be drawn.

### 3.4 Emulsion Stabilization

Stabilization of water-in-oil emulsions was considered at last, as this provides an applied demonstration of the newly synthesized surfactants. To be in line with interfacial tension measurements, the same petroleum distillate was tested at varied lignin concentrations and salinity. Right after emulsion preparation, the vials were visually checked to confirm that all of the water was finely emulsified. After overnight storage, some degree of coalescence was noted since large water droplets were visible within the bulk of droplets. Centrifugation was hence applied to collect the coalesced water at the bottom. In addition, the centrifugation likely promoted coalescence, which further contributed to free water. The nature of the emulsion was also confirmed by adding a few droplets of a densely packed emulsion to each of the two phases. In each tested case, the droplets would disperse well in the oil phase, indicating that this was a water-in-oil type of emulsion.

**Figure 7:**
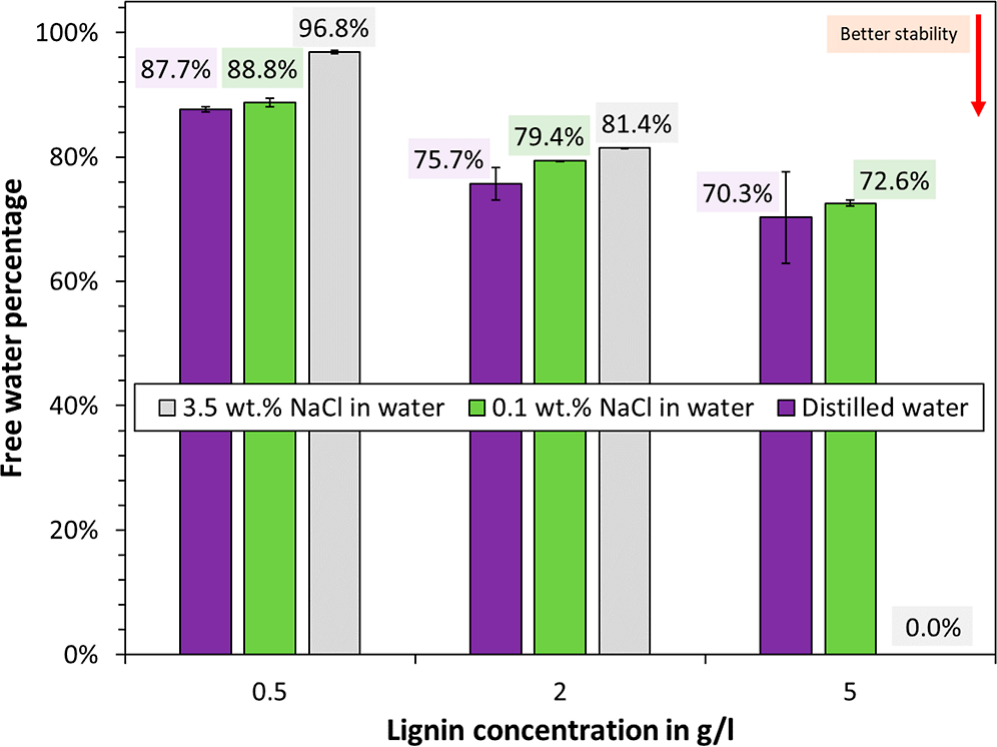
Stability of water in oil emulsions in dependence of salinity (NaCl in water) and concentration C_18_ esterified Kraft lignin in petroleum distillate.

The results are plotted in Figure 7. More free water correlated with worse emulsion stability, as a larger volume of droplets was able to undergo coalescence. As can be seen, an increase in the lignin concentration also yielded an increase in stability. This is consistent as interfacial adsorption is increased by elevating the concentration. In addition, bulk depletion effects tend to have a lower effect. Increasing the salinity decreased the emulsion stability at lignin concentrations of 2 and 0.5 g/L. It is unlikely that a decrease in Debye length contributed to this destabilization, as the dielectric constant in aliphatic oil is negligibly small. Increasing salinity is known to promote counterion condensation, which can reduce repulsive effects between ionized moieties in the lignin.^19^ As a result, the packing density at the interface can be increased, which tends to improve the stability of water-in-oil emulsions.^22,24^ This concept is likely also applicable to the oil-soluble lignin as part of the macromolecule will extend into the aqueous phase at the interface. Ionizable moieties, such as carboxylic acid groups, likely remained on the modified lignin, as only the hydroxyl groups were targeted by chemical modification. Still, it is curious to note that the opposite effect was achieved, i.e., destabilization at increased salinity. One outlier was noted, which is emulsions stabilized by 5 g/L C_18_ esterified Kraft lignin at 3.5 wt % NaCl salinity. Here, the emulsions were repeatedly so stable that no free water was obtained after centrifugation. The opposite has indeed been shown for water-soluble lignin surfactants, i.e., a destabilization at high salinity due to agglomeration and precipitation of the lignin.^22^ Again, the opposite trend was noted for the oil-soluble lignin in this study; yet, it appears that electrostatic interaction at the interface plays a role.

**Figure 8:**
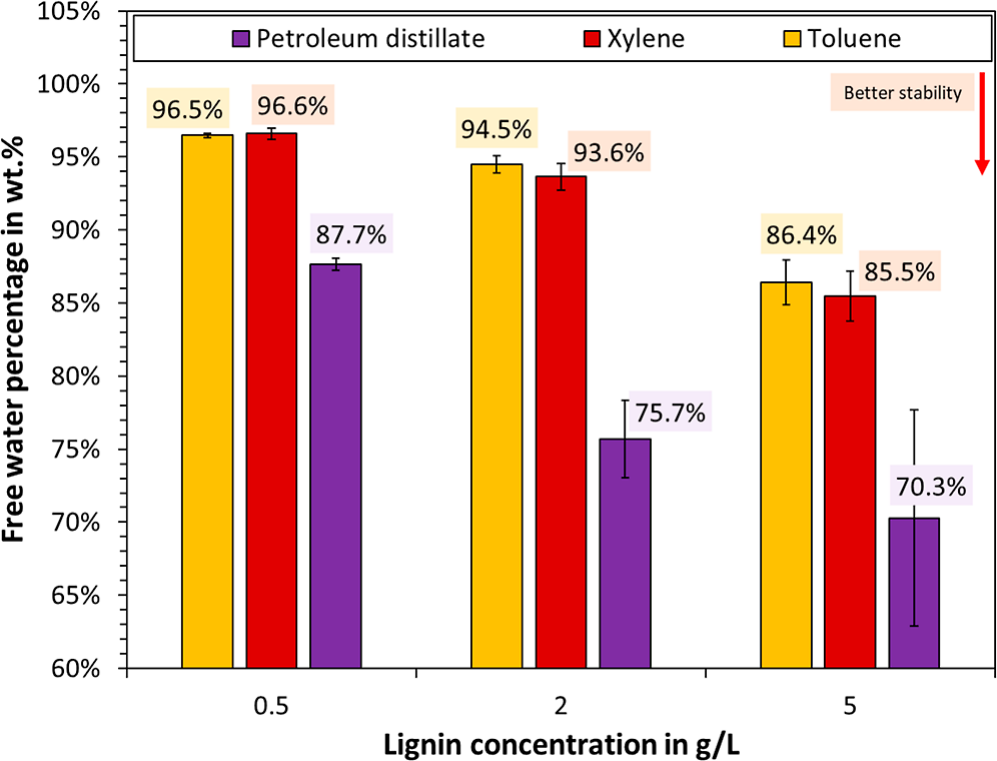
Stability of water in oil emulsions in dependence of oil phase and concentration of C_18_ esterified Kraft lignin.

At last, variations of the oil phase were made, which included the aromatic oils xylene and toluene as well as crude oil. The results are plotted in Figure 8. A lower stability was noted for the aromatic oils compared to that of the petroleum distillate. Judging by the speed and completeness of dissolution, the C_18_ esterified Kraft lignin exhibited better solubility in aromatic solvents than in aliphatic ones. For example, it was not possible to prepare solutions of the modified lignin in *n*-decane, as part of the sample remained as insoluble solids at 1 g/L. According to the supplier, the petroleum distillate contained only low amounts of aromatics. It is therefore plausible that petroleum distillate was a worse solvent than xylene or toluene. This would in turn drive the interfacial adsorption, as stabilization agents tend to perform best when these are on the verge of precipitation.^67^ Another explanation could be given in terms of the hydrophobic–lipophilic balance (HLB), i.e., the modified lignin exhibiting an HLB that more closely matched the water-in-petroleum distillate emulsions, as opposed to water-in-toluene or -xylene.

## 4 Conclusions

Lignin valorization and the development of biobased specialty chemicals are key areas, whose advancement can contribute to the development of green technologies. This article hence explored a new type of lignin-based surfactant that used esterification with fatty acids to render the lignin oil-soluble. This surfactant was furthermore characterized and tested for the effect on interfacial tension and suitability to stabilize water-in-oil emulsions.

Successful alkylation was indirectly confirmed by the lignin having obtained solubility in toluene, xylene, MeTHF, and a petroleum distillate. In addition, UV/vis spectrophotometry showed both a reduction in absorptivity and a hypsochromic shift (blueshift). The latter was attributed to blocking of the phenolic hydroxyl groups by esterification. Concurring with this were the FTIR measurements, which showed a decrease in OH-stretching, whereas the signals for CH-stretching and C=O stretching were significantly increased. At last, SEC showed an increase in polydispersity of the esterified Kraft lignin, which was attributed to the reaction and purification treatment. Compared to the butyrated Kraft lignin, the fatty acid grafts also had a higher apparent mass-average molecular weight.

Adsorption of lignin at the water–oil interface followed the same behavior as that for water-soluble lignin surfactants. A steep decrease in interfacial tension was noted at the start, followed by plateauing over the course of several hours. Low changes at the end of each measurement can be attributed to diffusional exchange and stearic reorientation at the interface. The equilibrated interfacial tension (*t* = 5 h) exhibited a linear logarithmic decrease at concentrations between 0.01 and 0.3 g/L. At higher concentrations, the change in the interfacial tension was lower, which suggested that aggregation to occur. A surface excess in the range of 7.5–9.0 ×10^–7^ mol/m^2^ and an area per molecule of 185–222 Å^2^ were calculated. The diffusion coefficient was decreasing at increasing concentration, which could be due to the lignin undergoing adsorption in the agglomerated state, the adsorption process not being purely diffusion-controlled, or stearic rearrangement at the interface. The data furthermore suggested that the alkyl-pendant chains of the oil-soluble lignin were extended outward into the solvent, as the diffusion coefficients of C_18_ esterified Kraft lignin were lower than for its C_12_ counterpart, albeit the latter having a higher apparent mass-average molecular weight. Emulsion stability tended to be better at high lignin concentrations and low salinity. Aromatic oils exhibited lower emulsion stability than the petroleum distillate, which was argued to arise from a lower solubility of alkylated lignin in the latter, hence driving the equilibrium toward greater adsorption.

Based on the results presented in this paper, Kraft lignin can be made oil-soluble by esterification with saturated fatty acids. This oil-soluble lignin further showed a surfactant-like behavior, mirroring the properties of water-soluble lignins. We therefore synthesized and characterized a novel type of lignin-based surfactant that may open up new application areas in oil-based systems. Future work may focus on determining and correlating the degree of esterification with the physicochemical properties of the esterified lignin, which can facilitate tailoring and fine-tuning for specific end-uses.
